# Photodetecting properties of single CuO–ZnO core–shell nanowires with p–n radial heterojunction

**DOI:** 10.1038/s41598-020-74963-4

**Published:** 2020-10-29

**Authors:** Andreea Costas, Camelia Florica, Nicoleta Preda, Andrei Kuncser, Ionut Enculescu

**Affiliations:** Multifunctional Materials and Structures Laboratory, Functional Nanostructures Group, National Institute of Materials Physics, 405A Atomistilor Street, 077125 Magurele, Ilfov Romania

**Keywords:** Nanowires, Electronic devices

## Abstract

CuO–ZnO core–shell radial heterojunction nanowire arrays were obtained by a simple route which implies two cost-effective methods: thermal oxidation in air for preparing CuO nanowire arrays, acting as a p-type core and RF magnetron sputtering for coating the surface of the CuO nanowires with a ZnO thin film, acting as a n-type shell. The morphological, structural, optical and compositional properties of the CuO–ZnO core–shell nanowire arrays were investigated. In order to analyse the electrical and photoelectrical properties of the metal oxide nanowires, single CuO and CuO–ZnO core–shell nanowires were contacted by employing electron beam lithography (EBL) and focused ion beam induced deposition (FIBID). The photoelectrical properties emphasize that the p–n radial heterojunction diodes based on single CuO–ZnO core–shell nanowires behave as photodetectors, evidencing a time-depending photoresponse under illumination at 520 nm and 405 nm wavelengths. The performance of the photodetector device was evaluated by assessing its key parameters: responsivity, external quantum efficiency and detectivity. The results highlighted that the obtained CuO–ZnO core–shell nanowires are emerging as potential building blocks for a next generation of photodetector devices.

## Introduction

Given the increasing demand for continuous miniaturization and reduced energy consumption, nanoscale optoelectronic devices based on semiconductor nanowires are emerging as an important novel class of devices with enhanced performances and improved or even new functionalities^1^. Due to their unique physico-chemical properties^2^, widespread research efforts are focused on the use of semiconductor nanowires as the new building blocks for functional devices in various fields which include optoelectronics^3^, biosensors^4^, spintronics^5^, etc. Nanowires are a particularly remarkable class of nanostructures owed to specific properties such as their one-dimensional geometry and large surface-to-volume ratio. These lead to an increase of the light confinement and photosensitivity^6^, features essential for applications such as photodetectors^7^, lasers^8^, solar cells^9^, photocatalysis^10^, etc. Additionally, the progress made in tuning the material properties during their synthesis led to the development of heterostructure nanowires with different architectures, such as axial, radial or branched^11–13^. Amidst them, radial core–shell heterostructure nanowires obtained using two semiconductors arranged in a type II (staggered gap) band alignment augmenting the charge carrier separation efficiency. In this type of heterojunction, charge separation region is distributed over the radius of the nanowire, which is significantly smaller than its length leading to an improved collection at the electrodes^14^. Thus, these radial core–shell heterostructure nanowires can be regarded as “smart materials” with controlled interfaces and enhanced multi-functionalities that can lead to the development of the next-generation high-performance optoelectronic devices^14–16^. Hence, radial core–shell nanowire arrays based on ZnO–Cu_x_O^16^, ZnO/CuCrO_2_^17^, ZnO/WS_2_^18^, ZnO–Cu_2_O^19^, ZnO–Co_3_O_4_^20^, ZnO/ZnS^21^, CdSe/ZnTe^22^, etc. with applications in photodetectors have been reported.

Copper oxide (CuO) is a p-type semiconductor with a narrow band gap in the range of 1.2–2.1 eV^23^, and high optical absorption in the visible range. It is also highly versatile and easy to prepare material, suitable for applications in field effect transistors^24^, photodetectors^25^, light emitting diodes^26^, solar cells^27^, photocatalysis^28^, etc. Zinc oxide (ZnO) is a n-type semiconductor with a wide band gap (3.37 eV), a 60 meV exciton binding energy^29^. Additionally, it is a relatively abundant and non-toxic material with a broad family of morphologies for its easy to prepare nanostructures (nanowires, nanotubes, rods, nanofibers, nanoflowers, etc.)^30–34^, finding applications in electronic devices^35^, sensors^36^, photodetectors^37^, solar cells^38^, photocatalysis^39^ etc. By combining the two materials, CuO and ZnO, into a radial core–shell architecture as CuO–ZnO nanowires, a type II band alignment is obtained between the two semiconductors. The radial heterostructure architecture leads to an improved charge carrier collection efficiency due to the formation of a specific, high surface area junction which separates charges with enhanced efficiency and suppresses their recombination^16,40^. Consequently, such radial core–shell heterostructure can lead to an enhancement of the photocurrent produced under illumination, fundamental in photodetectors^14^, these optoelectronic devices being in the spotlight of the researchers due to their applications in a wide range of areas like advanced communications devices, flame detectors, ozone sensors, missile warning system devices, healthcare related sensors, etc.^7,41,42^.

Hitherto, only few studies were reported on the preparation of the CuO–ZnO radial core–shell heterostructure nanowires having CuO nanowires as core and ZnO thin film layer as shell confirming the advantages of the combination of these two metal oxides in optoelectronic devices^43–46^. In these works, the CuO nanowire arrays were obtained by thermal oxidation in air^43–45^ or by merging photolithography, e-beam evaporation and thermal oxidation in air^46^ while the ZnO thin film layer was deposited by thermal decomposition^43–45^ or by atomic layer deposition^46^. Such CuO–ZnO core–shell array structures give the possibility to measure a collective photoresponse from all the nanowires in the array. Thus, a 10 mm wide × 20 mm long × 0.5 mm high copper foil entirely covered with CuO–ZnO nanowires exhibits a rise and decay time of 4.2 s and 5.2 s and a photocurrent gain of 0.264 mA under white light illumination^45^. However, single CuO–ZnO core–shell radial heterojunction nanowires based photodetectors were not reported to date, these devices giving the possibility to evaluate the photodetecting properties of the p–n radial heterojunction itself, without the influence coming from the other nanowires or from electrodes covering multiple nanowires. Moreover, such architecture is well suited for photodetection applications, with a shell window layer (the semiconductor with a wider band gap) and a core absorbing layer (the semiconductor with a narrower bandgap).

Considering all the aspects mentioned above and taking into account the key features provided by the geometry of the radial heterostructure architecture and by the type II band alignment that can be obtained between CuO and ZnO, analysing the photodetecting properties of single CuO–ZnO core shell nanowires could lead to the fabrication of photodetectors with enhanced performances suitable in a wide range of applications.

In this work, we demonstrate a facile route to obtain p–n junction heterostructures consisting in CuO–ZnO core–shell nanowire arrays, further these nanowires being successfully characterized as single photodetecting elements. Thus, the CuO–ZnO core–shell nanowire arrays were prepared by a two-step path, thermal oxidation in air of copper foils for the CuO core and radio frequency magnetron sputtering for depositing the ZnO shell. Thermal oxidation in air is a simple and cost-effective approach to prepare nanowire arrays at a rapid large-scale production without the use of hazardous and expensive chemicals^47^. Furthermore, the diameter and the length of the nanowires can be tuned by modifying the substrate temperature, growth time or substrate thickness^24,47^. Radio-frequency magnetron sputtering is a well-established thin film deposition technique used at industrial scale to obtain homogeneous strongly adhesive thin films on substrates with complex geometries^48^. The properties of the prepared CuO nanowire arrays and CuO–ZnO core–shell nanowire arrays were thoroughly analysed from morphological, structural, compositional and optical point of view.

Further, for evaluating the electrical and photoelectrical properties of the single CuO–ZnO core–shell nanowires, these were contacted using photolithography, electron beam lithography (EBL) and focused ion beam induced deposition (FIBID). Hence, the ZnO shell was contacted at one end using Ti/Au electrode and the CuO core was contacted at the other end of the nanowire using Pt. Under light exposure of the radial heterojunction, through the n-type semiconductor (ZnO shell), electron–hole pairs are photogenerated that are split at the interface with the p-type semiconductor (CuO core). Due to the radial geometry, the charges can be collected at the electrodes with a higher efficiency owed to the small diameter of the single core–shell nanowire, compared to the length of the nanowire. Besides, the obtained Ohmic contact on each end of the single CuO–ZnO nanowire, maximize the charges collection at the electrodes. Consequently, the novel aspect evidenced by this study includes the photodetecting properties of single CuO–ZnO core–shell nanowires contacted by lithographic techniques. It was therefore shown that the p–n radial heterojunction diode based on a single core–shell nanowire can be employed as a high performance, ultra-miniaturized photodetector. Additionally, a novel approach based on the ZnO dissolution in water-based solutions was used at the nanoscale dimensions, to ensure Ohmic contacts to both the core and the shell of the nanowire.

## Materials and methods

### Materials

All chemical reagents were acquired from Merck and used without further purification. Deionized water was obtained using a Millipore system. The copper foils were purchased from Alfa Aesar Thermo Fisher Scientific. The zinc oxide (99.999% purity), platinum (99.99% purity) and titanium (99.995% purity) sputtering targets, and the gold wire (99.99% purity) were bought from Kurt J. Lesker Company Ltd. (UK).

### Preparation of CuO nanowires and CuO–ZnO core–shell nanowire arrays

**Figure 1:**
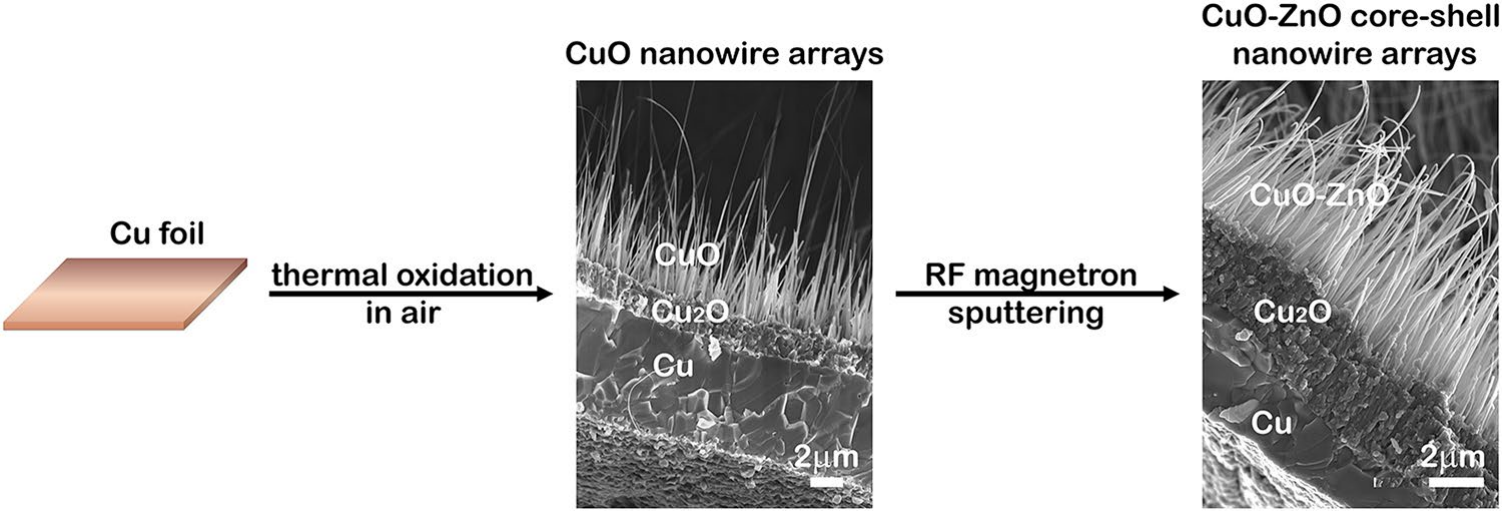
Schematic representation of the main stages involved in the preparation of the CuO–ZnO core–shell nanowire arrays.

The CuO nanowire arrays were prepared using a simple and high throughput method, thermal oxidation in air, in accordance with a previous work^24^. Firstly, copper foils of 1 mm thickness were cut into 2 cm^2^ slices each and washed for 5 min in acetone and isopropyl alcohol using an ultrasonic bath from Elma Schmidbauer GmbH. Then, the copper foils were rinsed several times in deionized water, dried under a nitrogen gas flow, placed on a ceramic substrate. Afterwards, the copper foils were thermally oxidized in air at a 450 °C temperature for 12 h at the ambient pressure in a convection oven from Nabertherm GmbH. In order to obtain the CuO–ZnO core–shell nanowire arrays, the surface of the CuO nanowire arrays was coated with a thin film of ZnO by radio-frequency (RF) magnetron sputtering technique using a Tectra GmbH Physikalische Instrumente equipment. A zinc oxide target having a diameter of 2 in. and a thickness of 0.125 in. was used in the deposition process, the following parameters being used: Ar atmosphere with a purity of 9.6 (99.9999%) as the working gas, the pressure in the sputtering chamber was 5.4 × 10^–3^ mbar, the RF power applied on the magnetron was 100 W, the deposition time was 10 and 20 min for tuning the thickness of the ZnO shell. Then, the CuO–ZnO core–shell nanowire arrays prepared on copper foils were transferred in ultrapure isopropyl alcohol by ultrasonication achieving a suspension of nanowires. The two type of CuO–ZnO core–shell nanowires with different ZnO shell thickness were labelled as CuO–ZnO_1 (10 min deposition) and CuO–ZnO_2 (20 min deposition). The main stages implied in the preparation of the CuO–ZnO core–shell nanowire arrays are illustrated in Fig. 1.

### Fabrication of p–n diodes based on single CuO–ZnO core–shell nanowires

**Figure 2:**
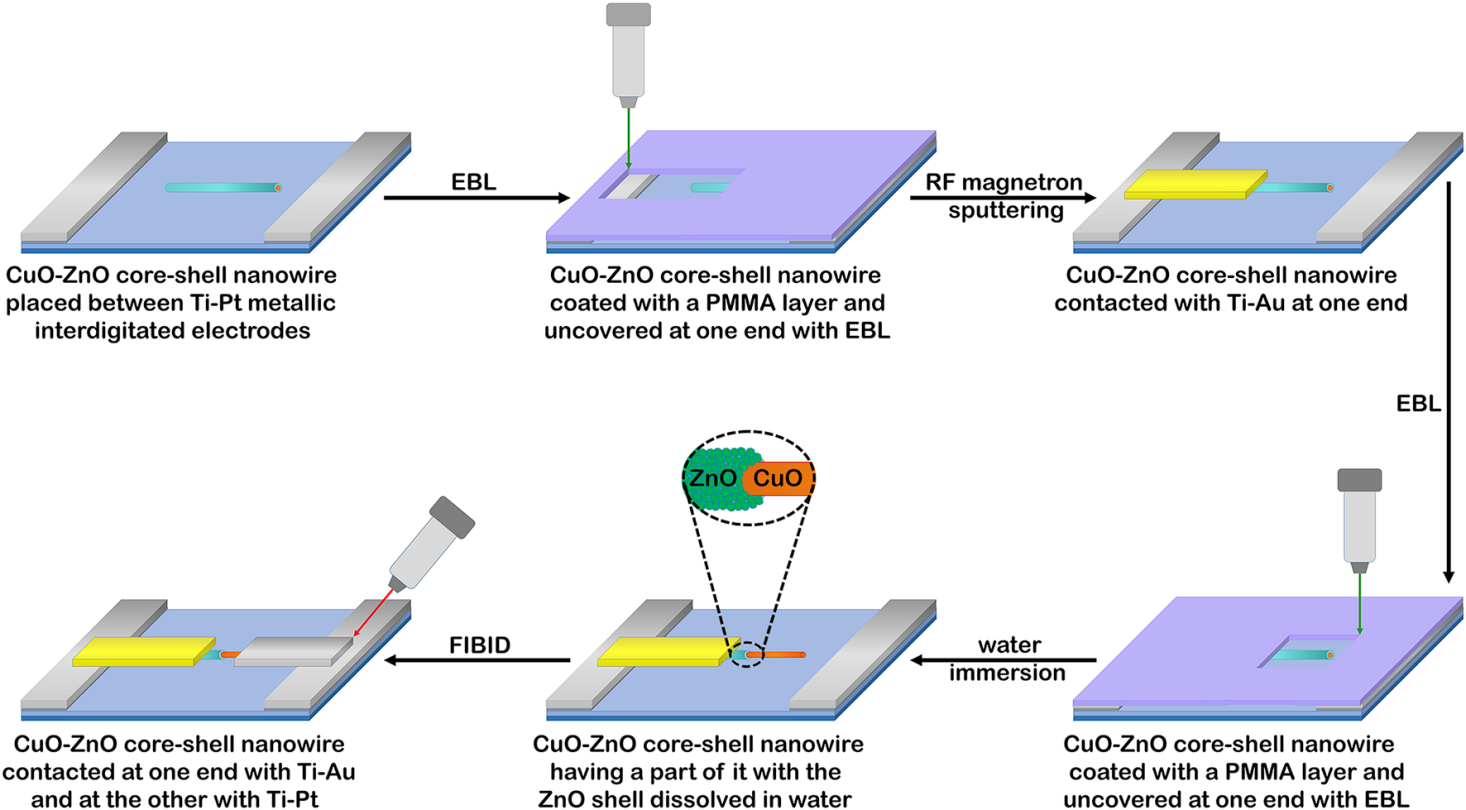
Schematic representation of the steps implicated in the fabrication of the electronic devices based on single CuO–ZnO radial heterojunction nanowires.

A schematic representation of the main steps implied in contacting single CuO–ZnO core–shell nanowires is given in Fig. 2. Thus, initially, Ti–Pt metallic interdigitated electrodes (the thicknesses was 10 nm for Ti and 100 nm for Pt) were fabricated on Si/SiO_2_ substrates (the thickness of SiO_2_ was 50 nm) by means of photolithography (EVG 620 Mask Alignment System) and RF magnetron sputtering (Tectra equipment). In the photolithography process, an UV sensitive resist acquired from MicroChemicals GmbH was used. Subsequently, droplets from the suspension of CuO–ZnO core–shell nanowires in isopropyl alcohol were dripped onto Si/SiO_2_ substrates patterned with Ti–Pt interdigitated electrodes. Taking into account that for CuO, Ohmic contacts are obtained with Pt^24^, and for ZnO Ohmic contacts are obtained with Ti–Au^30^, in order to develop single nanowire based photodetectors, single CuO–ZnO core–shell nanowires must have as contacts at one end of the nanowire Ti–Au and at the other end of the nanowire Pt to emphasize the properties of the radial heterojunctions. Consequently, electron beam lithography (Raith nanolithography system with Zeiss Merlin field emission scanning electron microscope) was used to design the Ti–Au contact connecting one end of the single CuO–ZnO core–shell nanowire with the Ti–Pt interdigitated electrodes using an electron beam sensitive resist, polymethyl methacrylate (PMMA) purchased from Micro resist technology GmbH. Further, the Ti–Au thin film electrode (the thicknesses was 100 nm for Ti and 200 nm for Au) was deposited by RF magnetron sputtering and thermal vacuum evaporation.

The next step was to dissolve a part of the ZnO shell at the other end of the single CuO–ZnO core–shell nanowire to be able to connect only the CuO core with a Pt contact that leads to the Ti–Pt interdigitated electrodes. In this way, comparing to our previous study^16^, proper electrical contacts are provided to the single p–n radial heterojunction nanowire diode. Thus, considering that ZnO at the nanoscale dimension can be dissolved in water-based solutions^39,49^, a second EBL process was performed for controlled opening of a well-defined area at the other end of the single CuO–ZnO core–shell nanowire in a new PMMA layer. Afterwards, the device was immersed for 30 min in a glass beaker containing deionized water, ZnO being removed from the exposed area. The final step was to connect the part of the CuO core exposed by ZnO dissolution to the Ti–Pt interdigitated electrodes by employing a Pt contact obtained by focused ion beam induced deposition (Tescan Lyra dual system FIB/SEM). Hence, the Pt layer, with a thickness of 300 nm, was deposited by FIBID using a Ga^+^ ion source at 150 pA ion beam current and an organometallic precursor (CH_3_)_3_Pt(CpCH_3_) (cyclopentadienyl trimethyl platinum). Usually, this type of deposition contains Pt metallic nanoparticles embedded in a carbon matrix with different levels of Ga implantation and oxygen contamination^50^, giving an Ohmic contact for CuO nanowires^24^.

All the lithographic systems (photolithography, EBL and FIBID) employed in the development of the optoelectronic devices based on single CuO–ZnO core–shell nanowires are located into a cleanroom facility ISO 5 and ISO 6.

### Material characterization

The morphology of the CuO nanowires and CuO–ZnO core–shell nanowire arrays was investigated with a field emission scanning electron microscope—FESEM (Zeiss Merlin Compact) and a high-resolution transmission electron microscope—TEM (Cs probe-corrected JEM ARM 200F analytical electron microscope). The crystalline structure of the nanowires was analysed with a X-ray diffractometer—XRD (Bruker AXS D8 Advance instrument with Cu Ka radiation, λ = 0.154 nm). The composition of the nanowires was identified by energy dispersive X-ray spectroscopy—EDX in TEM. The optical properties of the nanowires were examined by optical reflectance spectroscopy (Perkin–Elmer Lambda 45 UV–VIS spectrophotometer equipped with an integrating sphere).

### Electrical and photoelectrical measurements

The electrical and photoelectrical properties of the UV photodetectors based on single CuO–ZnO core–shell nanowires were studied in the ambient temperature and pressure using a probe station (Keithley 4200 SCS and a Cascade Microtech MPS 150), a Siglent SPD3303S source and laser diode modules with wavelengths of 405 nm, 520 nm and 635 nm from Laser Components GmbH. The laser diode modules were aligned perpendicular to contacted the single nanowires.

## Results and discussion

### Morphological, structural and optical properties

**Figure 3:**
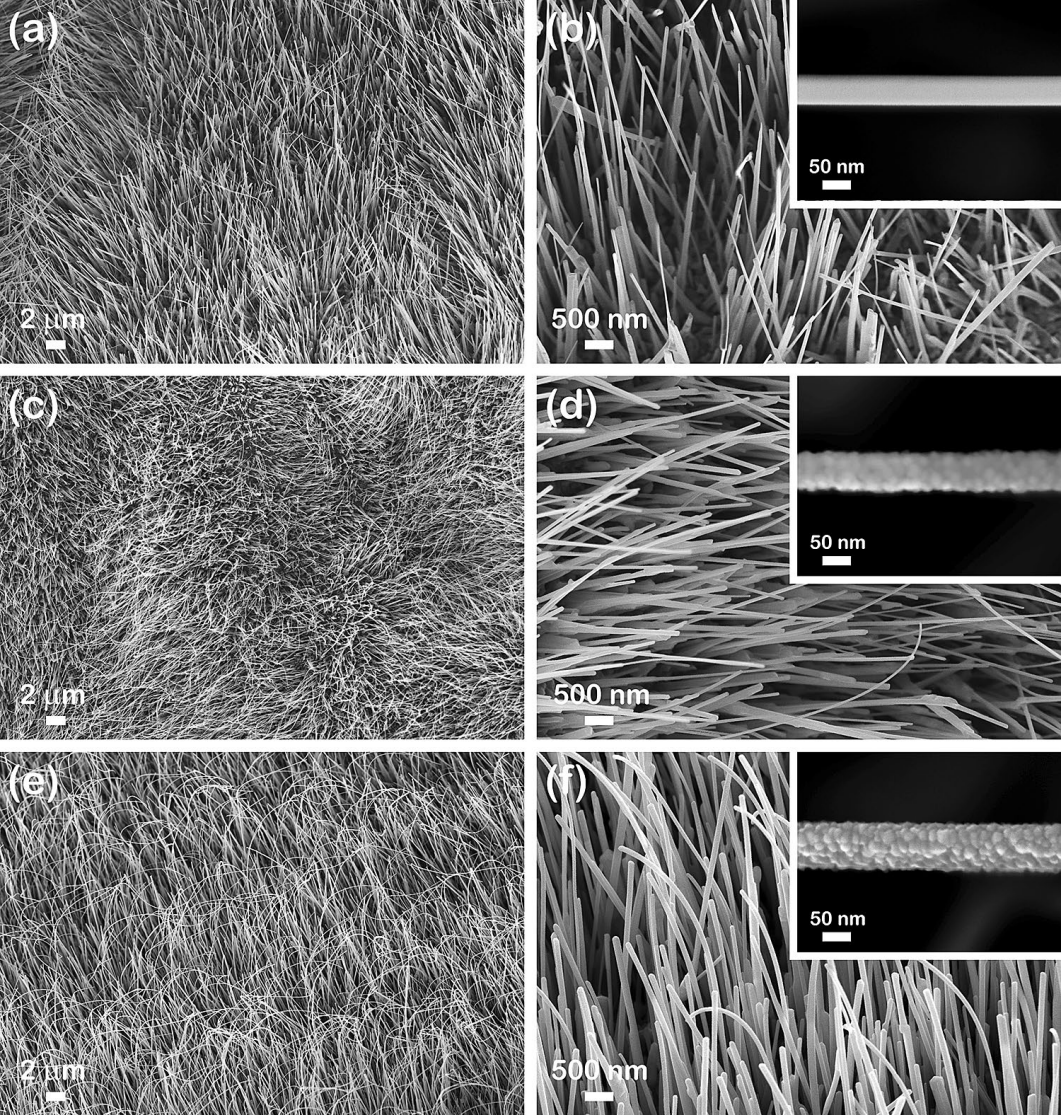
FESEM images at different magnifications of: (**a**, **b**) CuO nanowire arrays, (**c**, **d**) CuO–ZnO_1 core–shell nanowire arrays and (**e**, **f**) CuO–ZnO_2 core–shell nanowire arrays. Insets: FESEM images of a single (**b**) CuO nanowire, (**d**) CuO–ZnO_1 core–shell nanowire and (**f**) CuO–ZnO_2 core–shell nanowire.

The morphological properties of the CuO nanowires and CuO–ZnO core–shell nanowire arrays are presented in Fig. 3. The FESEM images at lower magnification of the CuO nanowires (Fig. 3a) and CuO–ZnO core–shell nanowire arrays (Fig. 3c,e) revealed that the nanowires have a typical cylindrical shape and a high aspect ratio, uniformly covering the Cu foil. Moreover, the CuO nanowires are vertically grown on the Cu foil, a slight tilt being observed in the case of the CuO–ZnO_2 core–shell nanowires due to the deposition of a thicker ZnO shell. The FESEM images at higher magnification of the CuO nanowires (Fig. 3b inset) and CuO–ZnO core–shell nanowire arrays (Fig. 3d inset,f inset) exhibit an average diameter of about 50 nm for the CuO nanowires, 70 nm for CuO–ZnO_1 core–shell nanowires and 80 nm for the CuO–ZnO_2 core–shell nanowires. Thus, the thickness of the ZnO shell can be estimated at around 10 nm for CuO–ZnO_1 and 15 nm for CuO–ZnO_1. In addition, it can be noticed that the ZnO shell covering the surface of the CuO nanowires consists in a granular nanostructured thin film.

**Figure 4:**
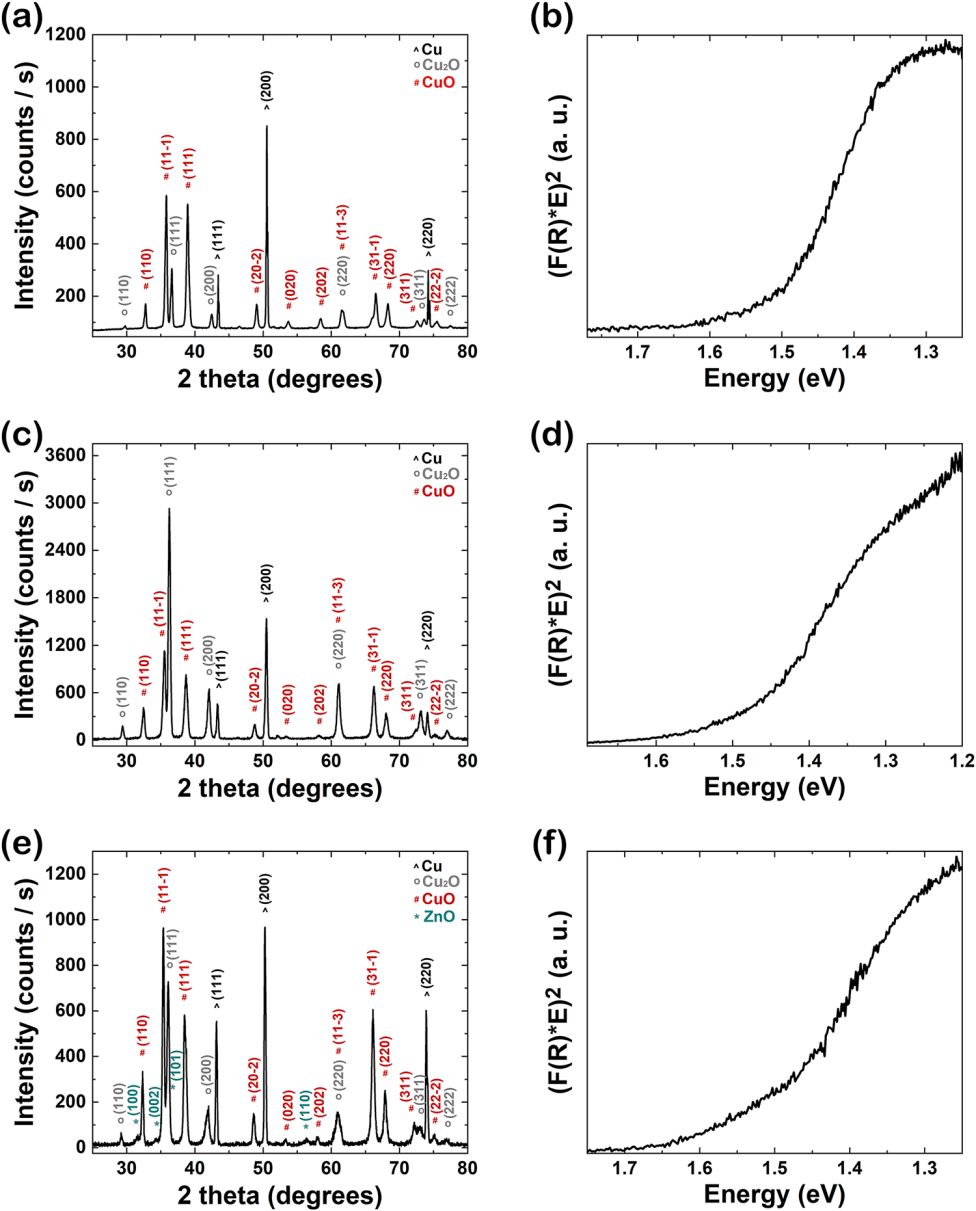
(**a**, **c**, **e**) XRD patterns and (**b**, **d**, **f**) Kubelka–Munk function representations used to estimate the band gap value for (**a**, **b**) CuO nanowire arrays, (**c**, **d**) CuO–ZnO_1 core–shell nanowire arrays and (**e**, **f**) CuO–ZnO_2 core–shell nanowire arrays.

The structural and optical properties of the nanowire arrays were performed on the as prepared Cu foil covered by CuO nanowires and CuO–ZnO core–shell nanowires and are given in Fig. 4. The XRD pattern of the CuO nanowires (Fig. 4a) reveals diffraction peaks at 2θ related to three phases: (1) 43.2°, 50.4° and 74.1° assigned to the Miller indexes of the reflecting planes for Cu in face-centred-cubic phase: (111), (200) and (220) (JCPDS reference code 00-004-0836), (2) 29.5°, 36.4°, 42.3°, 61.3°, 73.5° and 77.3° corresponding to the Miller indexes of the reflecting planes for Cu_2_O in cubic phase: (110), (111), (200), (220), (311) and (222) (JCPDS reference code 01-071-3645) and (3) 32.5°, 35.5°, 38.7°, 48.7°, 53.4°, 58.2°, 61.5°, 66.2°, 68.1°, 72.3° and 75.2° attributed to the Miller indexes of the reflecting planes for CuO in monoclinic phase: (110), (11$\overline{1}$), (111), (20$\overline{2}$), (020), (202), (11$\overline{3}$), (31$\overline{1}$), (220), (311) and (22$\overline{2}$) (JCPDS reference code 00-048-1548). The presence of these three phases in the XRD pattern of the CuO nanowires is also sustained by the cross-sectional FESEM image of the Cu foil covered with CuO nanowires (Fig. 1) in which it can be clearly observed three different regions with specific morphologies: the Cu foil at the base coated by a Cu_2_O film which is uniformly covered by CuO nanowire arrays.

The XRD pattern of the as prepared CuO–ZnO_1 core–shell nanowire arrays (Fig. 4c) shows only the phases related to the Cu, Cu_2_O and CuO, most probably due to the thickness of the ZnO shell (~ 10 nm). The XRD pattern of the CuO–ZnO_2 core–shell nanowires (Fig. 4e) evidences, additionally to the diffraction maxima attributed to Cu foil, Cu_2_O film and the CuO nanowires, peaks at 2θ: 31.7°, 34.4°, 36.2° and 56.6° corresponding to the Miller indexes of the reflecting planes for ZnO in hexagonal wurtzite phase: (100), (002), (101) and (110) (JCPDS reference code 00-036-1451) proving the presence of the ZnO shell.

The band gap value for the CuO nanowires and CuO–ZnO core–shell nanowire arrays were evaluated based on the reflectance data using Kubelka–Munk representation. Thus, by plotting (F(R)*E)^2^ versus the photon energy (E) for CuO nanowires (Fig. 4b), CuO–ZnO_1 core–shell nanowires (Fig. 4d) and CuO–ZnO_2 core–shell nanowires (Fig. 4f), where F(R) is the Kubelka–Munk function, with F(R) = (1 − R)^2^/2R and R the measured diffuse reflectance, the band gap value was estimated at ~ 1.5 ± 0.1 eV. The band gap values are consistent with those previously reported for CuO nanowires^51^ and CuO–ZnO core–shell heterostructures^52^.

**Figure 5:**
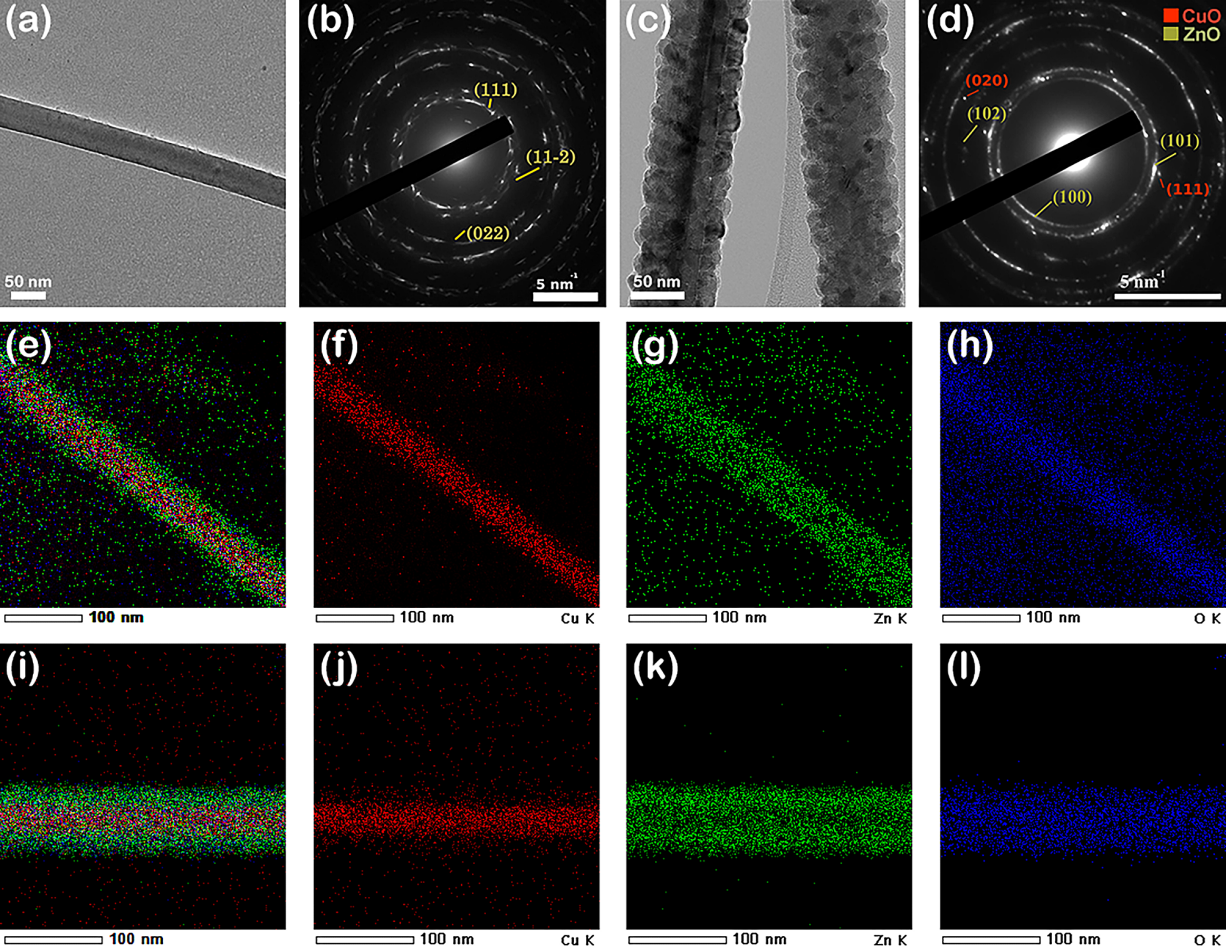
(**a**) TEM image and (**b**) SAED pattern of a single CuO nanowire, (**c**) TEM image and (**d**) SAED pattern of a single CuO–ZnO_2 core–shell nanowire, (**e**–**l**) EDX elemental mapping of a single CuO–ZnO core–shell nanowires and of the Cu, Zn and O as individual elements for (**e**–**h**) CuO–ZnO_1 and (**i**–**l**) CuO–ZnO_2.

Further, in order to emphasize the core–shell heterostructure, the single CuO nanowires and CuO–ZnO core–shell nanowires were investigated by TEM, the measurements being performed on TEM grides covered by drop casted CuO nanowires and CuO–ZnO core–shell nanowires from a suspension of nanowires in ultrapure isopropyl alcohol. Hence, the TEM image of a single CuO nanowire given in Fig. 5a evidences a smooth cylindrical shape and a diameter of 50 nm in agreement with the FESEM images (Fig. 3b, inset). A corresponding SAED pattern (Fig. 5b) confirms that the CuO nanowires have a poly-crystalline monoclinic structure in accordance with the XRD data (Fig. 4a). The TEM image of a single CuO–ZnO_2 core–shell nanowire (Fig. 5c) reveals that the cylindrical shape of the CuO nanowire core is preserved after the coating with the ZnO shell. Additionally, it can be noticed that the CuO core has a diameter of about 50 nm and the entire CuO–ZnO core–shell nanowire diameter is of about 80 nm, the thickness of the ZnO shell being evaluated at ~ 15 nm, confirming the data from the FESEM images from Fig. 3b, inset and Fig. 3f inset, respectively. The SAED pattern (Fig. 5d) of the CuO–ZnO_2 core–shell nanowires exhibits both components poly-crystalline structures, monoclinic for the CuO core and hexagonal wurtzite for the ZnO shell. Furthermore, Fig. 5e–l display the EDX elemental mappings of single CuO–ZnO_1 (Fig. 5e–h) and CuO_ZnO_2 (Fig. 5i–l) core–shell nanowires together with the EDX elemental mappings of Cu K, Zn K and O K as individual elements, emphasizing that the single CuO–ZnO core–shell nanowires are assembled only by these three elements. In addition, it can be observed the spatial distribution of these elements along the core–shell nanowires: the Cu K is present only in the core area, the Zn K being up to the surface area and the O K being uniformly distributed along the Cu K and Zn K areas.

### Photoelectric properties

**Figure 6:**
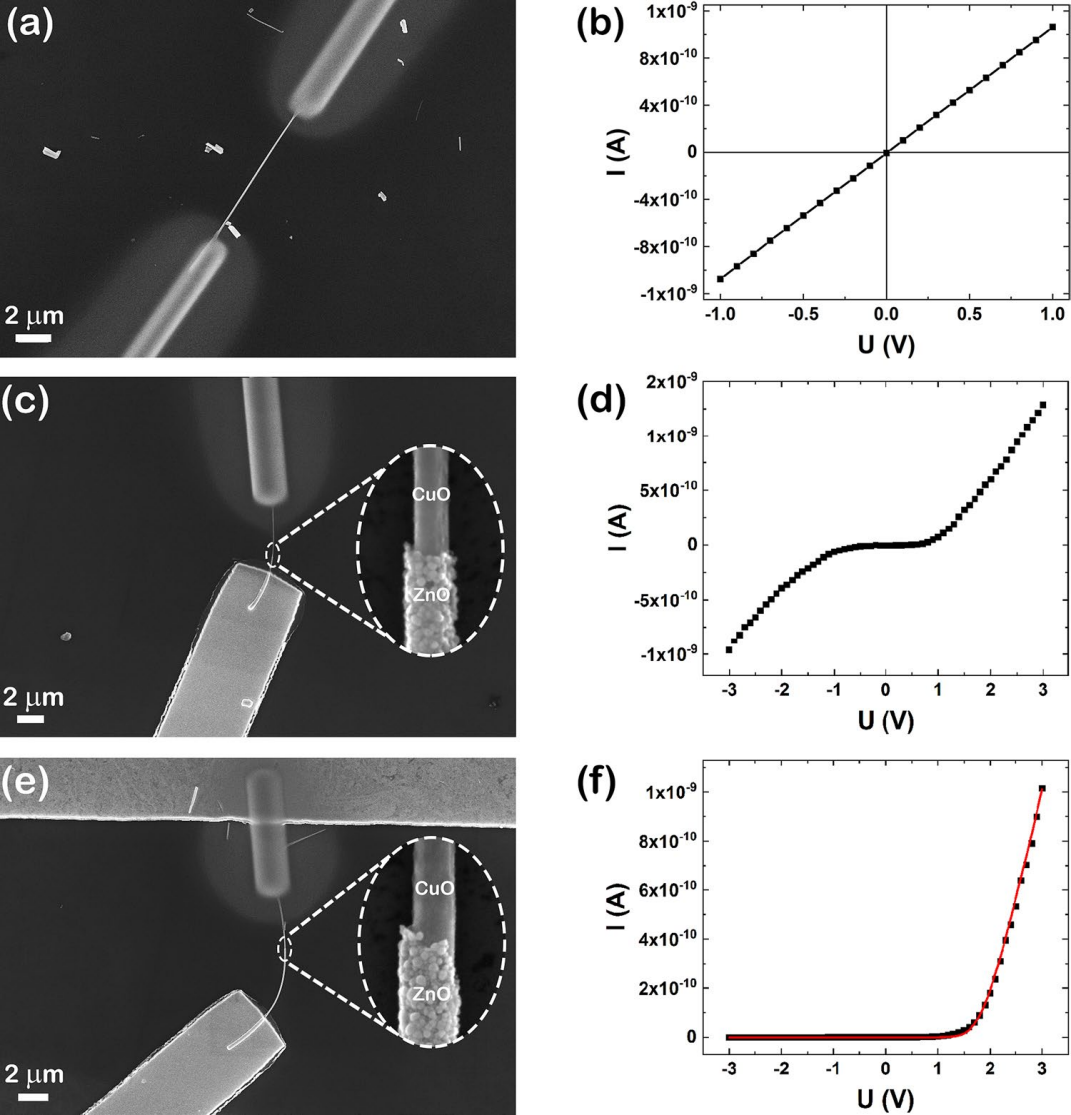
(**a**) FESEM image and (**b**) current–voltage characteristic of a single CuO nanowire contacted by FIBID, (**c**, **e**) FESEM images and (**d**, **f**) current–voltage characteristics of a single (**c**, **d**) CuO–ZnO_1 and (**e**, **f**) CuO–ZnO_2 core–shell nanowire contacted by EBL and FIBID. (red line—theoretical fitting). Insets: FESEM image at a higher magnification of the CuO–ZnO core–shell nanowires.

In order to assess the electrical and photoelectrical properties of the single CuO nanowires and CuO–ZnO core–shell nanowires, EBL and FIBID were employed to contact single nanowires. Thus, single CuO nanowires were contacted by FIBID with Pt contacts (thickness of 300 nm) from the two ends of the nanowire to the Ti–Pt interdigitated electrodes. Figure 6a presents a FESEM image of a single CuO nanowire contacted by FIBID with a distance of about 10 µm between the two Pt metallic contacts. Accordingly, single CuO–ZnO core–shell nanowires were contacted combining EBL, ZnO dissolution in water-based solutions at the nanoscale dimensions, FIBID and thin film deposition techniques resulting in single nanowires contacted at one end (on the ZnO shell) with Ti–Au (thickness of 100 nm/200 nm) using EBL and at the other end (on the CuO core) with Pt (thickness of 300 nm) using FIBID. Figure 6c shows a FESEM image of a single CuO–ZnO_1 core–shell nanowire contacted by EBL and FIBID with a distance of about 5 µm between the two Ti–Au and Pt metallic contacts, while Fig. 6e reveals a FESEM image of a single CuO–ZnO_2 core–shell nanowire with a distance between the contacts of about 9 µm. Moreover, the FESEM images at higher magnification (Fig. 6c inset,e inset) of the single CuO–ZnO_1 and CuO–ZnO_2 core–shell nanowires evidence the interface between CuO and ZnO obtained by the dissolution in water-based solution of the ZnO shell at one end of the CuO–ZnO core–shell nanowire. This allows the exploration of the electrical properties of the radial heterostructure itself, without accountable interference coming from the semiconductor/electrode interface when the thickness of the shell is appropriate.

The electrical measurements were performed at room temperature and ambient pressure in a conventional 2-points configuration. The current–voltage characteristic of a single CuO nanowire contacted by FIBID (Fig. 6b) displays a linear shape indicating the formation of Ohmic contacts between the CuO nanowire and the Pt contacts deposited by FIBID, in accordance with previously reported data^24^.

The current–voltage characteristic of a single CuO–ZnO_1 core–shell nanowire contacted by EBL and FIBID (Fig. 6d) evidences a non-linear symmetrical shape indicating a system of two back-to-back diodes connected to each other through a series resistance^53^. The result can be explained taking into account a direct contact between the Ti–Au and CuO core due to the diffusion of the metallic contacts through the thin ZnO shell giving rise to a second diode, added to the p–n heterostructure. The current–voltage characteristic of a single CuO–ZnO_2 core–shell nanowire contacted by EBL and FIBID (Fig. 6f) exhibits a non-linear asymmetric shape indicating a rectifying behaviour emerging from the formation of a p–n radial heterojunction between the CuO core and the ZnO shell of the single CuO–ZnO core–shell nanowire, typical for a p–n diode^54^. The semilogarithmic representation for the single CuO–ZnO_2 core–shell nanowire (Figure S1) reveals a direct–reverse ratio of about 10^3^ and the ideality factor of the p–n radial heterojunction diode based on this nanowire was estimated at about 1.5. Also, a theoretical fitting for the current–voltage characteristic of the p–n radial heterojunction diode based on single CuO–ZnO_2 core–shell nanowire was performed in order to determine its characteristic parameters. Thus, we considered a non-ideal p–n diode with an equivalent circuit model (Figure S2) formed by an ideal diode with a parasitic series resistance and a parallel shunt resistance^55^. The series resistance (R_S_) represents the resistance of a single CuO–ZnO core–shell nanowire and the parallel shunt resistance (R_sh_) is attributed to the metallic/semiconductor interfaces formed between the metallic contacts and the core–shell nanowire: Pt/CuO core and Au–Ti/ZnO shell. Hence, the equation depicting the current flow through the p–n radial heterojunction diode is:

1 $$I={I}_{S}\left\{exp\left[\frac{U\left(1+\frac{{R}_{S}}{{R}_{sh}}\right)-I{R}_{S}}{n{U}_{T}}\right]-1\right\}+ \frac{U}{{R}_{sh}}$$

where, I is the current flow throughout the p–n diode, I_S_ is the junction reverse saturation current, U is the applied voltage, R_S_ is the series resistance, R_sh_ is the parallel shunt resistance, n is the ideality factor and U_T_ = kT/q is the thermal voltage. For obtaining an analytical solution for this equation we used Lambert W function, representing the solution of $W{\mathrm{e}}^{\mathrm{W}}=\mathrm{x}$ equation^55^. Consequently, based on Lambert W function, the analytical solution for Eq. (1) is:

2 $$I=\frac{n{U}_{T}}{{R}_{s}}W\left[\frac{{I}_{S}{R}_{s}}{n{U}_{T}}{e}^{\left(\frac{U+{I}_{S}{R}_{s}}{n{U}_{T}}\right)}\right]-{I}_{S}+ \frac{U}{{R}_{sh}}$$

Based on Eq. (2) for the suggested equivalent circuit model, the current–voltage characteristic of the p–n radial heterojunction diode based on a single CuO–ZnO_2 core–shell nanowire (Fig. 6f) was fitted (Fig. 6f, red line). Thus, performing the fitting of the experimental data with the current given by the equivalent circuit, characteristic parameters for the p–n radial heterojunction diode based on the single CuO–ZnO_2 core–shell nanowire were estimated: n = 1.47, I_S_ = 3.43 × 10^–12^ A, R_S_ = 2.23 × 10^7^ Ω, and R_sh_ = 3.57 × 10^9^ Ω. Hence, comparing the obtained data for the direct-reverse ratio (10^3^) and for the ideality factor (1.5), the specific parameters are in accordance with data reported in the literature for p–n diodes based on single nanowires^56,57^.

**Figure 7:**
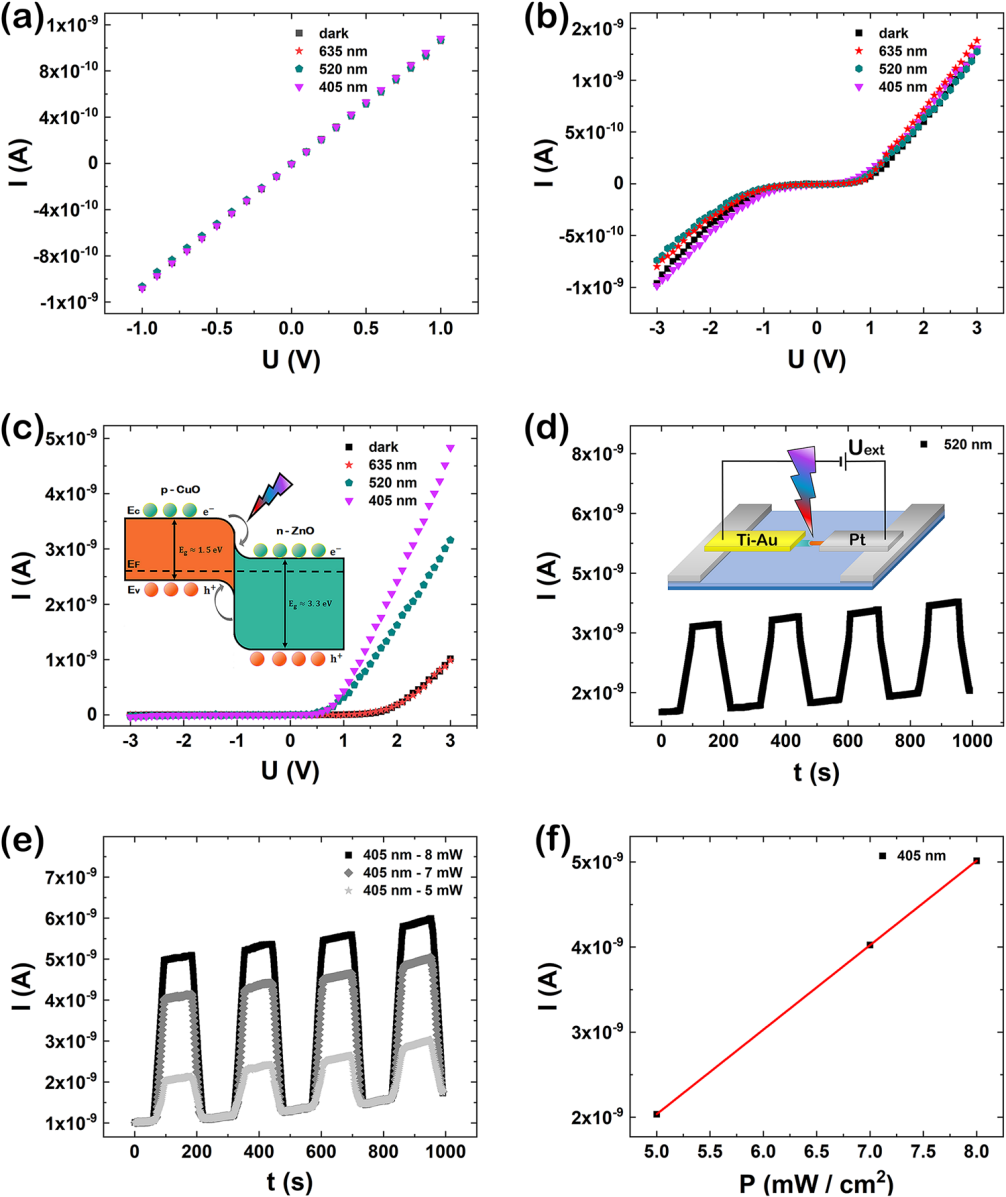
Current–voltage characteristics of a single (**a**) CuO nanowire, (**b**) CuO–ZnO_1 and (**c**) CuO–ZnO_2 core shell nanowire, under dark (black squares) and under illumination at 635 nm (red stars), 520 nm (turquoise hexagons) and 405 nm (magenta triangles) wavelengths. Current–time characteristic at (**d**) 520 nm and (**e**) 405 nm (at different applied power) of a p–n CuO–ZnO_2 diode. (**f**) Photocurrent as a function of the input laser power at 405 nm and 3 V bias voltage for the p–n CuO–ZnO_2 diode. Insets: (**c**) schematic representations of the band diagram CuO–ZnO heterojunction and (**d**) the contacted single CuO–ZnO core–shell nanowire under illumination.

The obtained p–n diodes based on single CuO–ZnO core–shell nanowire are promising candidates for application in photodetectors due to the radial heterojunction architecture formed between the CuO core and the ZnO shell which will determine an enhancement of the photocurrent under illumination. Therefore, in order to test the fabricated CuO–ZnO core–shell nanowire based p–n radial heterojunction diodes as photodetectors, photoelectric measurements were carried out. For comparison reason, photoelectrical measurements were also performed on single CuO nanowires contacted by FIBID. Thus, the current–voltage characteristics of single CuO nanowire contacted by FIBID, CuO–ZnO_1 and CuO–ZnO_2 core–shell nanowire contacted by EBL and FIBID are presented in Fig. 7a–c, respectively, the measurements being carried out in dark (black squares) and under illumination with different wavelengths: 635 nm (red stars), 520 nm (turquoise hexagons) and 405 nm (magenta triangles) in forward and reverse bias. In the case of single CuO nanowire (Fig. 7a) and CuO–ZnO_1 core–shell nanowire (Fig. 7b), regardless the wavelength applied, no significant photocurrent is noted, the current–voltage characteristics of the contacted nanowires preserving their initial shape in both dark and under illumination conditions. For single CuO–ZnO_2 core–shell nanowire (Fig. 7c), a non-linear asymmetrical shape attributed to the radial heterojunction formed between the CuO core and the ZnO shell is emphasized. In this case, there is no increase in the photocurrent under illumination at 635 nm wavelength while an increase in the photocurrent can be easily remarked under illumination at 520 nm and 405 nm wavelengths. The increase in the photocurrent of the p–n radial heterojunction diode results due to the combination of CuO core and the ZnO shell into a staggered gap heterojunction which leads in an enhancement of the charge separation at the CuO–ZnO core–shell interface^40,58^.

A schematic representation of the band diagram for a CuO–ZnO heterojunction under illumination, obtained in a p–n diode based on a single CuO–ZnO_2 core–shell nanowire is depicted in Fig. 7c, inset. Under illumination of the p–n CuO–ZnO heterojunction, the generated photocurrent is enhanced due to the type II band alignment obtained in the radial core–shell architecture by suppressing the recombination of the photogenerated charges and improving the photogenerated charge carrier collection efficiency^40,58^. Figure 7d,e evidence the time dependent photoresponse at a bias of 3 V of a single CuO–ZnO_2 core–shell nanowire-based photodetector under illumination at 520 nm and 405 nm, respectively.

It can be noticed a rise and decay time of 35 s for both wavelengths and a photocurrent gain of 2.23 nA at 520 nm and 4.01 nA at 405 nm. Comparing to our previous study^16^, it can be remarked and improvement in terms of photocurrent gain and rise and decay time at 405 nm, most probably due to the improved geometry of the single CuO–ZnO core–shell nanowire contacted with Pt directly on the CuO core and with Ti–Au on the ZnO shell. Furthermore, it was observed an increase of the photocurrent in time under illumination due to the effect of heating produced through the illumination. Figure 7d inset reveals a schematic representation of a single CuO–ZnO_2 core–shell nanowire contacted by EBL and FIBID, under illumination. In addition, a linear dependency can be observed for the photocurrent as a function of the input laser power characteristic at 405 nm and 3 V bias voltage (Fig. 7f), confirming the photodetector behavior for the single CuO–ZnO_2 core–shell nanowires contacted by EBL and FIBID. The expected response at wavelengths close to UV is given by the architecture of the heterojunction itself, due to the photogenerated electrons that can easily pass the barrier towards the conduction band. An infrared wavelength photogenerates holes that need to surpass a much higher barrier towards the valence band.

In order to evaluate the performance of the fabricated photodetector based on a single CuO–ZnO_2 core–shell nanowire, the specific parameters for a photodetector like responsivity (R_λ_), external quantum efficiency (EQE) and detectivity (D^*^) were determined using the following equations^59^:

3 $${R}_{\lambda }=\frac{\Delta I}{PS}$$

4 $$EQE={R}_{\lambda }\frac{hc}{e\lambda }$$

5 $${D}^{*}=\frac{{R}_{\lambda }}{\sqrt{\frac{2e{I}_{dark}}{S}}}$$

where, λ is the light wavelength, ΔI represents the difference between photocurrent and dark current (I_dark_), S is the effective illuminated area, P represents the incident light power, c is the speed of light, e is the elementary charge and h is the Planck constant. Using these equations, the key parameters of a photodetector (R_λ_, EQE and D^*^) were estimated to be: 4.84 A/W, 11.59% and 22.52 × 10^9^ Jones for 520 nm and 8.74 A/W, 26.56% and 40.5 × 10^9^ Jones for 405 nm, respectively. The values obtained for the R_λ_, EQE and D^*^ parameters are in agreement with data reported in the literature for photodetectors based on core–shell nanowires^16,59–61^.

## Conclusions

Arrays of CuO–ZnO core–shell nanowires architectures were prepared using thermal oxidation in air in order to obtain CuO nanowires (core) and RF magnetron sputtering for coating the CuO nanowires with a thin film of ZnO (shell). The morphological characterization reveals that the CuO–ZnO core–shell nanowire arrays have a high aspect ratio with diameters of about 80 nm and a thickness of the ZnO shell estimated at ~ 10 nm for CuO–ZnO_1 and ~ 15 nm for CuO–ZnO_2. The structural investigation evidences that the CuO nanowires have a monoclinic structure and the ZnO thin film has a hexagonal wurtzite structure. From the optical measurements, a band gap of ~ 1.5 ± 0.1 eV was estimated for all the investigated nanowire arrays. In order to assess the electrical and photoelectrical properties of the nanowires, single CuO nanowires and CuO–ZnO core–shell nanowires were contacted by means of EBL and FIBID. To contact single CuO–ZnO nanowires, a novel approach was used, based on the ZnO dissolution in water-based solutions at the nanoscale dimensions, to ensure Ohmic contacts to both the core and the shell of the nanowire. The electrical properties of the single nanowires exhibit an Ohmic behaviour for the CuO nanowire, a back-to-back diode for the CuO–ZnO_1 nanowire and a rectifying p–n diode behaviour for the CuO–ZnO_2 nanowire. Analysing the p–n radial heterojunction diode based on single CuO–ZnO_2 core–shell nanowires, the characteristic parameters of the diodes were estimated: a direct-reverse ratio of about 10^3^ and an ideality factor of about 1.5. The photoelectrical properties reveal that the p–n radial heterojunction diode based on a single CuO–ZnO_2 core–shell nanowire can be employed as a photodetector. Moreover, the key parameters (responsivity, external quantum efficiency and detectivity) of a photodetector based on a single CuO–ZnO_2 core–shell nanowire were estimated to be: 4.84 A/W, 11.59% and 22.52 × 10^9^ Jones for 520 nm and 8.74 A/W, 26.56% and 40.5 × 10^9^ Jones for 405 nm, respectively. The results proved that these core–shell heterostructure architectures having a type II band alignment formed at the p-type CuO core—n-type ZnO shell interface lead to an enhancement of the photodetector performances, promoting the CuO–ZnO core–shell nanowires as the new building blocks for the next generation ultra-miniaturized photodetector devices.

## Supplementary information

Supplementary Figures.

## Data Availability

The datasets supporting the conclusions of the current study are presented in the manuscript and supporting information.
