# Supplementary material for: Photodetecting properties of single CuO–ZnO core–shell nanowires with p–n radial heterojunction
Source: Sci Rep. 2020 Oct 29;10:18690. doi: 10.1038/s41598-020-74963-4 (PMC7596234; doi:10.1038/s41598-020-74963-4)
Supplement: Supplementary file 1 — Supplementary Figures. [file 41598_2020_74963_MOESM1_ESM.docx]

Photodetecting properties of single CuO-ZnO

core-shell nanowires with p-n radial heterojunction

*Andreea Costas^#*^, Camelia Florica^#**^, Nicoleta Preda, Andrei Kuncser & Ionut Enculescu^***^*

National Institute of Materials Physics, Multifunctional Materials and Structures Laboratory, Functional Nanostructures Group, 405A Atomistilor Street, 077125, Magurele, Ilfov, Romania

^#^Andreea Costas and Camelia Florica contributed equally to this work.

^*^Corresponding author: [andreea.costas@infim.ro](mailto:andreea.costas@infim.ro) (A. Costas)

^**^Corresponding author: [camelia.florica@infim.ro](mailto:camelia.florica@infim.ro) (C. Florica)

^***^Corresponding author: [encu@infim.ro](mailto:encu@infim.ro) (I. Enculescu)





Figure S1. Semilogarithmic representation of the current-voltage characteristic of an individual CuO-ZnO_2 core-shell nanowire, contacted by means of EBL and FIBID.


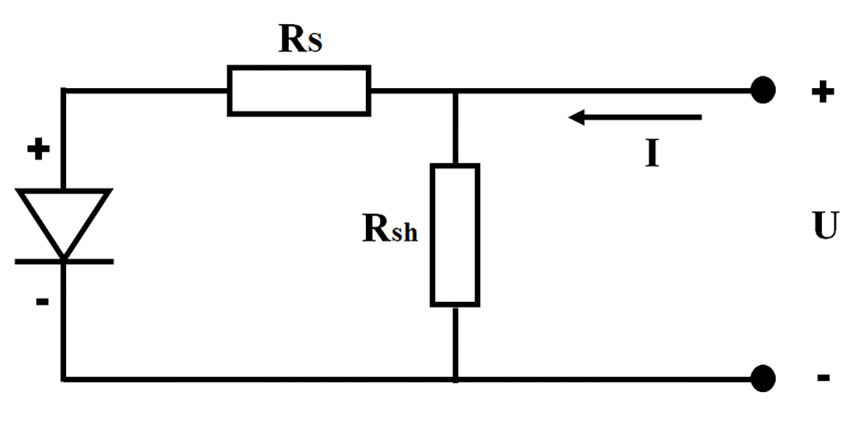


Figure S2. Equivalent circuit model consisting in an ideal diode,

a parasitic series resistance and a parallel shunt resistance.
